# The Role of Saikosaponins in Therapeutic Strategies for Age-Related Diseases

**DOI:** 10.1155/2018/8275256

**Published:** 2018-04-12

**Authors:** Byeong Mo Kim

**Affiliations:** Severance Integrative Research Institute for Cerebral & Cardiovascular Diseases (SIRIC), Yonsei University College of Medicine, 50 Yonsei-ro, Seodaemun-gu, Seoul 03722, Republic of Korea

## Abstract

As life expectancy increases, elderly populations tend to spend an increasing number of years in poor health, with chronic age-related diseases and disability. Therefore, the development of therapeutic strategies to treat or prevent multiple pathophysiological conditions in the elderly may improve health-adjusted life expectancy and alleviate the potential economic and social burdens arising from age-related diseases. Bioactive natural products might represent promising new drug candidates for the treatment of many chronic age-related diseases, including cancer, Alzheimer's disease, cardiovascular disease, obesity, and liver disease. Here, we discuss a therapeutic option using saikosaponins, which are triterpene saponins isolated from *Bupleurum*, against a variety of age-related diseases. Understanding the underlying mechanisms of natural products like saikosaponins in the treatment of age-related diseases may help in the development of diverse natural product-derived compounds that may be effective against a number of chronic health problems.

## 1. Introduction

As the average lifespan increases, as a result of improved living standards, there is greater concern regarding health-adjusted life expectancy. Unfortunately, health-adjusted life expectancy has not reached longevity, which means the prevalence of age-related diseases such as cancer, Alzheimer's disease (AD), cardiovascular disease, diabetes, and liver disease is likely to increase in the future. These diseases usually cause physiologic deterioration and resultant morbidity and mortality at an advanced age. Therefore, therapeutic strategies to intervene in chronic age-related diseases are important for achieving a healthy old age. With the aim of prolonging healthy aging or postponing biological aging, a variety of approaches are being used to develop effective methods for the treatment or prevention of age-related diseases. Because aging and age-related disease are believed to go hand in hand, drugs and/or trials to treat or prevent chronic age-related diseases might extend health-adjusted life expectancy. Oxidative stress and inflammation are involved in the common pathological features of age-related diseases.

Natural products can play an important role in the development of new drugs as they may have advantages over conventional chemical compound-based medications, such as fewer side effects, less long-term toxicity, variable bioavailability, and unidentified chemical structures and biological activities. Bioactive natural products are derived from various sources including plants, animals, marine organisms, and microorganisms. Plant-derived natural products have been the primary materials for the treatment of diseases. Almost every part of the plant, including the leaf, flower, bark, pod, fruit, seed, and root, can be exploited for drug development. Natural products can be classified as alkaloids, carbohydrates, glycosides, terpenoids, steroids, and phenolics. Saikosaponins are triterpene saponin glycosides derived from the traditional oriental medicinal plant, *Bupleuri Radix*, which has been widely used for its antioxidant, anti-inflammatory, antipyretic, and antihepatotoxic effects in the treatment of influenza, fever, hepatitis, malaria, and menstrual disorders [1–5]. Previous research has shown that saikosaponins exhibit a variety of pharmacological activities including antioxidant, anti-inflammatory, antibacterial, antiviral, and anticancer effects [6–10]. To date, more than 100 saikosaponins have been identified.

**Figure 1:**
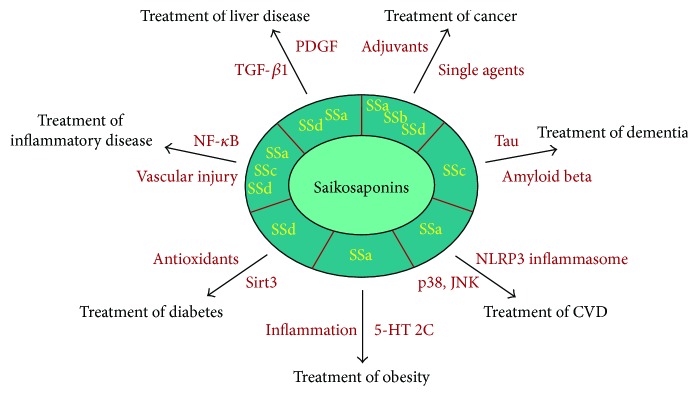
The use of saikosaponins and implications for age-related diseases. Several types of saikosaponins, such as saikosaponin a (SSa), saikosaponin b (SSb), saikosaponin c (SSc), and saikosaponin d (SSd), could be used to treat age-related diseases. SSa, SSb, and SSd may be used for the treatment of cancer as single anticancer or adjuvant agents. Inhibitory actions of saikosaponins against P-glycoprotein-mediated multidrug resistance are implicated in the ability of saikosaponins to sensitize cancer cells to chemotherapy or radiotherapy. SSc has dual effects on human Alzheimer's disease by targeting both tau and amyloid beta. SSa may slow or reverse the progression of atherosclerosis by inhibiting oxidized low-density lipoprotein-induced activation of p38 and the c-Jun N-terminal kinase mitogen-activated protein kinase pathway and assembly of the NLR family pyrin domain containing 3 inflammasome. SSa exerts antiobesity activity by acting as a 5-hydroxytryptamine 2C receptor agonist or by suppressing extracellular signal-regulated kinase/nuclear factor- (NF-) *κ*B pathway-induced inflammation. SSd provides protection against diabetic nephropathy through the upregulation of Sirt3 followed by mitochondrial antioxidant enzymes like isocitrate dehydrogenase 2 and manganese-dependent superoxide dismutase. SSa, SSc, and SSd may be used for the treatment of inflammatory disorders, such as asthma, arthritis, and sepsis, by downregulating the NF-*κ*B signaling pathway or inhibiting vascular injury/apoptosis. Lastly, SSa and SSd protect the liver against age-associated injury and fibrosis by suppressing cytokines, including platelet-derived growth factor and transforming growth factor-*β*1.

Although natural product-based drug development is expected to be helpful for the prevention and treatment of various chronic degenerative diseases, there is insufficient information on the underlying mechanisms of natural products in the body. In this review, we discuss the therapeutic implications of saikosaponins for the treatment of different types of age-related diseases (Figure 1). Due to their relatively profound founding and publishment, we discuss the therapeutic implications of the use of saikosaponins in cancer treatment in greater detail.

## 2. Therapeutic Approaches for Cancer

Cancer is the leading cause of death worldwide. Over several decades, many natural product-based drugs have been developed for cancer treatment, making up more than 60% of anticancer agents. Natural product-based anticancer agents can be used as a single agent or as an adjuvant with chemotherapy or radiotherapy. Among the saikosaponins, saikosaponin a (SSa), saikosaponin b (SSb), and saikosaponin d (SSd) have been shown to be anticancer agents. Compared with their anticancer activity, the possible role of saikosaponins in combating carcinogen-induced carcinogenesis has not been well studied. However, the protective effect of SSd on diethylinitrosamine-induced liver carcinogenesis has been reported [11]. Diethylinitrosamine-treated Sprague Dawley rats show histopathological features of putative preneoplastic lesions, such as liver nodule formation, tumor invasion of nearby organs, and increased cellular atypia. Interestingly, intraperitoneal injection of SSd remarkably reduced these lesions. SSd also lowers the expression of cyclooxygenase-2 (Cox-2) and CCAAT/enhancer binding protein *β* (C/EBP*β*), both of which are induced by diethylinitrosamine. These results suggest that SSd prevents diethylinitrosamine-induced liver carcinogenesis in rats via the downregulation of Cox-2 and C/EBP*β* and that SSd might be a novel chemopreventive agent against chemical-induced liver carcinogenesis.

SSa is a major bioactive compound of saikosaponins. SSa-induced anticancer activity has been well reported [12–19]. SSa has been shown to inhibit hepatoma cell growth and DNA synthesis [12, 14–16]. Given that mRNA and protein expression of the cyclin-dependent kinase (Cdk) inhibitors p15 and p16, but not of the Cdk inhibitors p21 or p27, are induced by SSa and that phosphorylation of extracellular signal-regulated kinase (Erk) is induced by SSa [15, 16], SSa-induced inhibition of hepatoma cell growth might be associated with the expression of p16 family proteins and the phosphorylation of Erk. Furthermore, pretreatment with PD98059, an inhibitor of MEK, an upstream kinase of Erk, prevents SSa-triggered inhibition of hepatoma cell growth and the accompanying induction of p15 and p16 [16], indicating that activation of Erk and the downstream induction of p16 family proteins are critical for SSa-induced inhibition of hepatoma growth. SSa also exhibits anticancer activity in breast cancer. SSa inhibits the proliferation or viability of human breast cancer cells by triggering apoptosis [17]. It induces apoptosis in MCF-7 (wild-type p53) cells through a p53-p21-dependent pathway and in MDA-MB-231 (mutant p53) cells through a p53-p21-independent pathway. In addition, SSa can induce the differentiation of rat C6 glioma cells into astrocytes and/or oligodendrocytes [13]. Our group has been investigating the therapeutic implications of SSa in colon cancer. We have found that SSa-induced cytotoxicity is greater in human colon cancer cells compared with other cancer cells, including lung cancer, breast cancer, and leukemia, indicating that SSa can trigger apoptotic cancer cell death in a cell type-dependent manner [19]. We also demonstrated that sequential activation of caspase-4, 2, and 8 is critical for SSa-induced caspase-3 activation and apoptosis in human colon cancer cells and that caspase-4 acts upstream of SSa-induced DNA damage [18, 19]. Given that SSa-induced apoptosis is specific to certain types of cancer, including colon cancer, care should be taken when SSa is used as a therapeutic tool in cancer treatment.

SSb is also a natural terpenoid. Among SSbs, saikosaponin b2 (SSb2) appears to exhibit anticancer activity, especially against melanoma. In B16 melanoma cells, a relatively high concentration of SSb2 (60–100 *μ*M) induces apoptosis, while chronic (30 days) low-dose SSb2 (5 *μ*M) induces differentiation rather than apoptosis [20, 21]. Interestingly, phorbol 12-myristate 13-acetate (PMA), a protein kinase C (PKC) activator, markedly inhibits both high-concentration SSb2-induced apoptosis and low-concentration SSb2-induced differentiation, suggesting that SSb2 exerts its anticancer activity by downregulating PKC activity. Given that a relatively high concentration of SSb2 is required to trigger apoptosis, SSb appears to be less effective against cancer cells compared with SSa and SSd.

SSd is also a triterpene saponin found in *Bupleurum* that exhibits a variety of pharmacological activities. Due to its diverse actions, a number of studies investigating SSd compared with other saikosaponins have been performed, especially in cancer research. The anticancer activity of SSd has been broadly investigated for a variety of cancer types, including hepatoma, glioma, melanoma, and lung, thyroid, and prostate cancers [12, 13, 22–31]. First, SSd exerts its anticancer effects against hepatoma cells through induction of caspase-3-dependent and independent apoptosis [22, 23], induction of p53-p21-mediated G1 phase arrest [23], downregulation of key regulators of cancer progression [25], and downregulation of the signal transducer and activator of transcription 3- (STAT3-) hypoxia inducible factor- (HIF-) 1*α*-Cox-2 pathway [29]. Second, like SSa, SSd can also induce the differentiation of rat C6 glioma cells into astrocytes [13]. However, unlike SSa, SSd cannot induce the differentiation of C6 glioma cells into oligodendrocytes. Third, SSd inhibits A549 lung cancer cell growth by inducing apoptosis and G1 phase arrest [24]. Enhanced expression of p53, p21, Bax, Fas, and Fas ligand appears to be the underlying mechanism of the anticancer effects of SSa in lung cancer, especially in nonsmall cell lung cancer. Fourth, SSd inhibits proliferation of human undifferentiated (anaplastic) thyroid carcinoma cells through p53-p21-mediated G1 phase arrest and Bcl-2 family-mediated apoptosis [27]. Fifth, SSd inhibits proliferation of human prostate cancer cells through p53-p21-mediated G1 phase arrest and Bcl-2 family-mediated mitochondrial apoptosis [28]. SSd also inhibits the growth and colony formation of prostate cancer cells by reversing the epithelial-mesenchymal transition and metastasis and by suppressing cancer stem cell phenotypes such as self-renewal [31]. Finally, SSd nanoparticles exhibit antimelanoma activity [30]. The activation of mitogen-activated protein kinases (MAPKs; p38 and c-Jun N-terminal kinase (JNK)) and p53 and cytosolic release of cytochrome *c* are involved in SSd nanoparticle-mediated antimelanoma activity. To date, SSd is the most potent compound with antimelanoma activity. SSd can also induce autophagic cell death, especially in apoptosis-defective cells. For example, SSd induced autophagy through the Ca^2+^-calcium/calmodulin-dependent protein kinase- (CAMKK-) AMP-activated protein kinase- (AMPK-) mammalian target of rapamycin (mTOR) pathway in HeLa and MCF-7 cells [26]. SSd can also cause the disruption of calcium homeostasis and resultant endoplasmic reticulum stress, representing another mechanism of autophagy induction in the same cancer cell lines. Therefore, SSd might also be a promising autophagy-regulating anticancer agent for targeting apoptosis-defective or apoptosis-resistant cancer cells.

The development of multidrug resistance (MDR) is a major obstacle in successful cancer chemotherapy. P-glycoprotein (P-gp), a transmembrane multidrug transporter capable of pumping many drugs out of cells and thus decreasing effective intracellular drug concentrations, is widely considered an important player in MDR. Both SSa and SSd appear to induce the reversal of P-gp-mediated MDR. SSa can increase the chemosensitivity of P-gp-overexpressing HepG2/adriamycin (ADR) and MCF-7/ADR MDR cancer cells to doxorubicin, vincristine, and paclitaxel by promoting apoptosis, increasing the retention of drugs, and reducing P-gp expression [32]. SSd can also increase the cytotoxicity of doxorubicin in MCF-7/ADR cells by inhibiting P-gp-mediated drug efflux, restoring drug accumulation, and downregulating P-gp expression [33]. Moreover, SSd can enhance the efficacy of doxorubicin in MCF-7/ADR xenograft mouse models as well as cell culture models without altering the pharmacokinetic profiles of doxorubicin [34]. Thus, both SSa and SSd may be further developed as potent reversal agents for P-gp-mediated MDR in cancer therapy.

Consistent with the ability of both SSa and SSd to reverse MDR in preclinical models, SSa and SSd might sensitize resistant cancer cells to chemotherapy or radiotherapy. Both SSa and SSd can sensitize various types of cancer cells, including cervical, ovarian, and nonsmall cell lung, to cisplatin-induced cell death [35]. SSa- and SSd-mediated sensitization is accompanied by an increase in reactive oxygen species (ROS) and an increase in caspase activation. Tumor necrosis factor- (TNF-) *α*, a multifunctional proinflammatory cytokine, can induce apoptotic cell death of certain types of cancer. However, TNF-*α*-induced activation of the transcription factor nuclear factor- (NF-) *κ*B and NF-*κ*B-driven expression of prosurvival factors is a major obstacle to preventing the cancer therapeutic efficacy of TNF-*α*. Therefore, inhibiting the activation of NF-*κ*B may improve the efficacy of TNF-*α*. SSd enhances TNF-*α*-mediated apoptosis by suppressing TNF-*α*-induced NF-*κ*B activation and the expression of its target prosurvival genes implicated in cancer cell proliferation, invasion, angiogenesis, and metastasis [36]. SSb can sensitize etoposide-induced apoptotic cell death in murine B16F10 melanoma cells by suppressing NF-*κ*B activation and inducing DNA damage [37]. The radiosensitizing activity of SSd has also been well reported in human hepatoma cells [38–40]. SSd increases the radiosensitivity of smmc-7721 hepatoma cells by inducing G0/G1 arrest in conditions of oxia and hypoxia [38]. SSd-mediated radiosensitization is accompanied by the upregulation of p53 and Bax and the downregulation of Bcl-2 in conditions of oxia and hypoxia [38, 39]. In particular, the SSd-mediated decrease in HIF-1*α* expression is critical for the effect of SSd on p53, Bax, and Bcl-2 in hypoxic conditions. SSd-mediated radiosensitization in hypoxia is also correlated with increased apoptosis [39]. Furthermore, the *in vivo* radiosensitizing activity of SSd was confirmed with subcutaneous smmc-7721 nude mouse xenografts [39]. SSd can also enhance radiation-induced DNA damage and, more importantly, upregulate the level of antioxidants following radiation treatment, indicating another beneficial effect of SSd [40]. Taking these findings into account, SSa and SSd can be used as adjuvant therapies together with chemotherapy or radiotherapy to enhance the therapeutic effect.

The structures of saikosaponins can be elucidated by comprehensive spectroscopic and chemical analysis. A novel spectroscopic approach has been developed to understand structure-activity relationships (SAR) more fully. SAR studies have shown that SSa and SSd, but not SSc, possess significant anti-inflammatory activities. In cancer therapy, SAR results suggest that the 13,28-epoxy bridge, in which C-28 can be a methylene, a hydroxymethylene, or a carbonyl group, is important for the selectivity and cytotoxicity of saikosaponins [41]. SAR studies also indicate that the spatial orientation of the hydroxyl group and the type of sugar unit are important for effective cancer therapeutic action by saikosaponins.

Although SSa, SSb, and SSd have been shown to exhibit antitumor activity, it is unlikely that all saikosaponins will be useful in cancer treatment. The results to date suggest that SSc is not suitable for cancer therapy, as it does not affect cancer cell growth. Instead, SSc may have the potential for therapeutic angiogenesis. For example, SSc induces human umbilical vein endothelial cell (HUVEC) growth, migration, and capillary tube formation [42]. Molecular signaling components, such as matrix metalloproteinase-2, vascular endothelial growth factor, and Erk, which are important in endothelial cell growth, migration, and angiogenesis, are also induced by SSc. We also found that SSc is less cytotoxic than other saikosaponins (unpublished data). Various approaches for using saikosaponins as a single modality or as an adjuvant for cancer therapeutic purposes are summarized in Tables 1 and 2, respectively.

## 3. Therapeutic Approaches for Dementia

Dementia is caused by disorders that affect brain function, such as memory, language, and learning. AD and vascular dementia are the most common types of dementia. AD is a progressive neurodegenerative disorder that causes the loss of intellectual and social skills including orientation, behavior, memory, thinking, and judgment. A combination of genetic, lifestyle and environmental factors influences the development and progression of AD. Brains from patients with AD are characterized by brain shrinkage (atrophy), which affects nearly all functions, including memory and thinking. The presence of tangles composed of phosphorylated tau and senile plaques composed of amyloid beta (A*β*) peptide, a metabolite of the sequential cleavage of amyloid precursor protein, is the neuropathological hallmark of AD.

Recently, we identified SSc as a potential natural compound to target both tau and A*β* [43]. Specifically, SSc inhibits the secretion of A*β*_1–40_ and A*β*_1–42_ and abnormal tau phosphorylation at multiple AD-related sites. Furthermore, SSc exerts beneficial effects on normal cellular tau function, accelerating nerve growth factor-mediated neurite outgrowth and promoting the assembly of microtubules. Together, these results indicate that SSc might be a novel therapeutic tool for treating human AD. The therapeutic activity of SSc in other forms of dementia, such as vascular dementia, has not been assessed.

## 4. Therapeutic Approaches for Cardiovascular Diseases

Cardiovascular diseases (CVDs) refer to a class of diseases that involve the heart and blood vessels, including coronary artery diseases (e.g., angina and myocardial infarction), stroke, heart failure, hypertensive heart disease, and other conditions affecting the cardiovascular system. The development of CVDs is associated with multiple risk factors. In East Asian countries, several natural products or derivatives have been used as traditional oriental herbal medicines to treat CVDs.

The therapeutic benefit of saikosaponins against atherosclerosis has been reported [44, 45]. Saikosaponins can inhibit oxidized low-density lipoprotein- (ox-LDL-) induced HUVEC injury and apoptosis by inhibiting the inflammatory response and oxidative stress [44]. Ox-LDL increases the level of inflammatory cytokines (TNF-*α* and interleukin- (IL-) 6), adhesion molecules (intercellular adhesion molecule-1 and vascular cell adhesion protein-1), and malondialdehyde (MDA) and reduces the activity of superoxide dismutase (SOD), all of which are significantly inhibited by saikosaponins. These antiatherosclerotic effects of saikosaponins are dependent on the inhibition of p38 and the JNK MAPK signaling pathway. Among the saikosaponins, SSa appears to be a major bioactive component involved in antiatherosclerosis activity. SSa attenuates ox-LDL-induced lipid uptake and promotes cholesterol efflux to inhibit foam cell formation, a hallmark of early atherosclerosis [45]. Concomitantly, SSa enhances the expression of ATP-binding cassette transporter A1 and peroxisome proliferator-activated receptor *γ*, both of which can protect against atherosclerosis and slow the progression of atherosclerosis. Mechanistically, SSa inhibits ox-LDL-induced activation of Akt and NF-*κ*B, assembly of the NLR family pyrin domain containing 3 (NLRP3) inflammasome, and the consequent release of inflammasome-dependent cytokines. These results suggest that SSa has therapeutic potential against atherosclerosis by modulating the PI3K/Akt pathway and the NLRP3 inflammasome.

Platelet aggregation induced by collagen, thrombin, and adenosine diphosphate (ADP) can lead to abnormal clotting and increase the risk of CVD such as heart attack and stroke via thrombotic and atherogenic mechanisms. Thromboxane is known to play an important role in platelet activation and coagulation following thrombin activation. SSa significantly inhibits ADP-induced platelet aggregation and platelet thromboxane formation from arachidonic acid [46]. The inhibitory effect of SSa on platelet activation indicates the therapeutic benefit of SSa in the maintenance of vascular homeostasis.

## 5. Therapeutic Approaches for Obesity

Obesity is a common and growing public health problem in modern life, and effective treatment is challenging. Obesity results in an increased mortality rate and significant health problems, including type 2 diabetes, hypertension, heart disease, stroke, hyperlipidemia, asthma, and certain types of cancer. However, the role of drug therapy in the comprehensive care of patients with obesity has been questioned. Most obesity drugs have both efficacy and safety issues. Long-term medication is limited in its effects on weight loss, and most patients regain weight once the use of obesity drugs is stopped. Abundant natural products have been explored for their potential to treat obesity, which could provide an excellent alternative strategy for the management of obesity.

Recently, the antiobesity action of saikosaponins has been reported [47, 48]. Modulating 5-hydroxytryptamine 2C (5-HT 2C) appears to be an important strategy for the treatment of obesity because the central 5-HT system is critical for the brain's control of energy homeostasis, and human obesity is associated with a diminished biosynthesis and chronic turnover of 5-HC in the brain. Saikosaponins exhibit agonistic activity on the 5-HT 2C receptor, thereby exerting antiobesity properties [47]. SSa appears to be the biologically active compound with antiobesity properties, with obvious agonistic activity on the 5-HT 2C receptor. SSa can also attenuate obesity-associated inflammation in hypertrophied 3T3-L1 adipocytes via the Erk/NF-*κ*B pathway [48]. Finally, SSa significantly decreases proinflammatory cytokines, such as TNF-*α* and IL-1*β*, and inflammatory factors, such as inducible nitric oxide synthase (iNOS) and Cox-2, by inhibiting the NF-*κ*B pathway in 3T3-L1 adipocytes. Given that pretreatment with U0126, an Erk inhibitor, inhibits nuclear translocation of NF-*κ*B, the antiobesity action of SSa appears to be mediated by reduced inflammation via the downregulation of Erk following NF-*κ*B inhibition.

## 6. Therapeutic Approaches for Diabetes

Diabetes mellitus, which is commonly referred to as diabetes, is a chronic metabolic disorder in which high blood glucose levels are present in the body over a prolonged period due to defects in the production and/or function of insulin. Although diabetes may not be a normal part of aging and type 1 diabetes can occur at any age, the incidence of type 2 diabetes, which is more common than type 1, increases with age and is usually seen in middle-aged or older individuals. Diabetes can damage both large and small blood vessels, thereby increasing the risk of heart attack, stroke, blindness, and kidney disease. In the treatment of diabetes, the efficacy of antidiabetics varies for each individual, indicating that more individual-specific diabetic treatment is needed. This may be because many antidiabetics work on only one or two causes of the disease. Side effects also present a significant problem as some antidiabetics cause side effects such as low blood glucose and/or weight gain.

Diabetes is a major risk factor for kidney disease because diabetes can damage the kidney. Diabetic kidney disease, also known as diabetic nephropathy, is the chronic loss of kidney function that occurs in diabetes mellitus over a patient's lifetime. SSd protects renal tubular epithelial cells against high glucose-induced diabetic nephropathy [49]. High glucose levels result in the production of highly reactive compounds (e.g., ROS and MDA), as well as a concomitant decrease in the activity of antioxidant enzymes (e.g., SOD) in renal tubular epithelial cells. Incubation with SSd significantly restores these changes via the upregulation of mitochondrial NAD-dependent deacetylase Sirt3 expression followed by the upregulation of the expression of mitochondrial NADP-dependent isocitrate dehydrogenase 2 and manganese-dependent SOD.

## 7. Therapeutic Approaches for Inflammation

Inflammation is triggered by the innate immune system as a defense mechanism against infection or tissue injury. However, the transition from transient and/or acute inflammation to chronic inflammation triggered by the dysregulation of the normal immune response leads to inflammatory disorders such as asthma, arthritis, and autoimmune diseases. Inflammation appears to be related to nearly every chronic disease, including cancer, dementia, cardiovascular diseases, and diabetes.

Saikosaponins are well known for their immunomodulatory antiallergic inflammation action [50]. SSd suppresses murine T lymphocyte activation [51], indicating that SSd may be a potential candidate for the treatment of autoreactive T lymphocyte-mediated autoimmune diseases. The inhibitory effects of SSd on PMA-triggered T lymphocyte activation are associated with SSd-induced downregulation of NF-*κ*B, nuclear factor of activated T-cells, and activator protein-1 (c-Fos) signaling pathways. SSd is also effective in the treatment of allergic reactions induced by *β*-conglycinin [52]. SSd inhibits *β*-conglycinin-induced activation and degranulation of rat basophilic leukemia-2H3 cells by suppressing intracellular mobilization and tyrosine phosphorylation followed by suppression of ROS generation, activation of Cdc42 and c-Fos, and *β*-hexosaminidase release. Because soybean *β*-conglycinins are potential food allergens, SSd might be an effective therapy for alleviating soybean allergy. Both SSa and SSd exhibit anti-inflammatory activity. iNOS and Cox-2 play pivotal roles in the development of certain inflammatory conditions, and modulating iNOS and Cox-2 is thought to be a promising therapeutic tool to inhibit inflammation. Both SSa and SSd inhibit the production of iNOS-derived nitric oxide and Cox-2-derived prostaglandin E2 triggered by lipopolysaccharide (LPS) in murine macrophage RAW264.7 cells [53]. The LPS-induced production of proinflammatory cytokines, such as TNF-*α* and IL-6, is also suppressed by SSa and SSd. All of the inhibitory effects of SSa and SSd have been attributed to the cytosolic retention of NF-*κ*B in RAW264.7 cells. Moreover, these saikosaponins have been shown to exhibit significant *in vivo* anti-inflammatory activity in two different murine models; a rat model of carrageenan-induced paw swelling and a mouse model of acetic acid-induced vascular permeability. Remarkably, these saikosaponins appear to be useful in the treatment of inflammatory diseases. For instance, SSa can exert inhibitory activity against a rat model of allergic asthma, a complex and chronic inflammatory disorder [54], and against inflammatory arthritis in a human osteoarthritis chondrocyte cell model [55]. These findings suggest that saikosaponins may be used in anti-inflammatory treatment strategies for chronic disease.

Sepsis, a life-threatening body-wide complication of infection, occurs through blood-borne infection with microorganisms such as bacteria, fungi, or viruses. Administration of small doses of bacterial LPS to mice triggers acute inflammation and endothelial damage and apoptosis, mimicking the early stages of septic shock in humans. We recently demonstrated that SSc inhibits LPS-induced apoptosis by inhibiting caspase-3 activation and the subsequent degradation of focal adhesion kinase in endothelial HUVECs [56]. Our results suggest that SSc represents a promising therapeutic candidate for the treatment of vascular endothelial cell injury and barrier dysfunction.

## 8. Therapeutic Approaches for Liver Disease

Consistent with the fact that aging affects the liver to a lesser degree than other organs and that there are no identified liver diseases specific to advanced age, there have been few comprehensive studies of liver degeneration during the aging process. Nonetheless, liver function decreases and liver damage increases with age. Acute liver injury has been shown to be greater in aged rats compared with younger rats [57, 58]. In a mouse liver model, age-related repression of three key regulators of liver function, C/EBP*α*, farnesoid X receptor, and telomerase reverse transcriptase, may lead to an increase in liver injury and apoptosis following carbon tetrachloride (CCl_4_) treatment [59]. Liver fibrosis is a consequence of the excessive accumulation of extracellular matrix (ECM) protein including collagen as a healing response triggered by chronic liver injury. Aging also increases susceptibility to hepatic inflammation and liver fibrosis and enhances the liver fibrotic response by disrupting ECM remodeling. Stellate cells are the major cell type involved in liver fibrosis. Following liver injury, hepatic stellate cells undergo activation, leading to increased proliferation and migration, which is responsible for the increased synthesis and deposition of ECM proteins in the liver and for amplification of the fibrotic response.

Although *Bupleurum* has yielded a number of pharmacologically active compounds, the most relevant for the treatment of liver disease is the saikosaponins. Both SSa and SSd have been shown to improve hepatic antioxidant capacity and protect against CCl_4_-induced liver injuries, such as inflammation and fibrosis, in rat models [8, 9, 60]. SSa and SSd also inhibit platelet-derived growth factor (PDGF) and transforming growth factor- (TGF-) *β*1-induced proliferation and migration of hepatic stellate cells [61]. Downregulation of Erk/PDGF/TGF-*β*1 signaling has been implicated in SSa- and SSd-mediated liver protection. Both SSa and SSd also significantly induce hepatic stellate cell apoptosis. Given that survival, proliferation, and migration of hepatic stellate cells are key drivers of liver fibrosis and ECM remodeling, SSa and SSd might exert therapeutic activities against liver fibrosis and damage by inhibiting the activation of hepatic stellate cells.

## 9. Conclusions and Perspectives

Natural products, such as herbal medicines, represent monotherapies or adjunctive therapies in the treatment of age-related diseases. Because conventional drugs have limitations and adverse side effects, the development of alternative medicines is necessary. Owing to the advantages of natural products, additional pharmaceutical agents will be discovered. Saikosaponins, which we have been studying for a long time, can be developed as novel potential drugs for future therapeutic strategies. Naturally occurring saikosaponins have been studied by different research groups and found to be very promising drug candidates for the prevention and treatment of various age-related diseases due to their multiple actions in inflammation, oxidant/antioxidant balance, and damage response.

However, despite its usefulness and potential for growth, natural product-derived medicine has not yet been widely applied in age-related diseases. As shown above, a significant portion of the research on natural products, including saikosaponins, has focused on cancer. It is still unknown whether saikosaponins exert therapeutic activity against other chronic disorders such as the forms of dementia other than AD and cardiovascular diseases other than atherosclerosis. Likewise, the therapeutic relevance of saikosaponins in the aging process remains unclear. Moreover, despite the extensive literature on the anticancer activities of saikosaponins, the cytotoxicity of saikosaponins has not been well investigated in normal cells. Therefore, it is necessary to evaluate the cytotoxic selectivity of saikosaponins for cancer cells versus normal cells. For research purposes, the accumulation of scientific data through cell culture, molecular biology, and *in vivo* mouse experiments will be necessary for an understanding of the underlying mechanisms of the therapeutic action and toxicity of natural products like saikosaponins in the body. Further studies of the possibly superior outcome of saikosaponin treatment over other components of *Bupleuri Radix* and the probable advantage of combined treatment with saikosaponin monomers and other components of *Bupleuri Radix* in the treatment of various age-related diseases are also required.

Saikosaponins are minimally soluble in water. Saikosaponins contain a rigid hydrophobic group and are dissolved in DMSO. Since DMSO may be toxic to normal cells, there are safety concerns regarding the use of DMSO as a solvent. However, given that less than 0.1% DMSO is generally noninfluential and that the final concentration of DMSO as a solvent of saikosaponins can be adjusted to be no greater than 0.1%, the possibility of DMSO toxicity in saikosaponin solution seems very slight. Nonetheless, the poor solubility of saikosaponins may be a major problem in future drug development. Therefore, various approaches to improve the solubility and bioavailability of saikosaponins should be considered. The application of pharmaceutical particle technologies, such as particle size reduction technologies and bioavailability enhancement technologies to saikosaponins may improve their solubility and pharmacokinetics for clinical development.

## Figures and Tables

**Table 1:** Therapeutic strategies for cancer using saikosaponins. Related references are listed.

Saikosaponins	Mode of action	Target molecules	Cancer model	Ref.
SSa	Growth inhibition	Not determined	Liver cancer	[12]
	Differentiation	Not determined	Glioma	[13]
	Increase in sub-G1 peak	Not determined	Liver cancer	[14]
	G1 phase arrest	p15, p16	Liver cancer	[15]
	G1 phase arrest	Erk, p15, p16	Liver cancer	[16]
	Apoptosis	Bcl-2, c-Myc, p53, p21	Breast cancer	[17]
	Apoptosis	Caspase-2, Caspase-8	Colon cancer	[18]
	Apoptosis, DNA damage	Caspase-4, Caspase-2	Colon cancer	[19]
SSb	G1 phase arrest	PKC	Melanoma	[20]
	Differentiation	PKC	Melanoma	[21]
SSd	Growth inhibition	Not determined	Liver cancer	[12]
	Differentiation	Not determined	Glioma	[13]
	Apoptosis	Not determined	Liver cancer	[22]
	G1 phase arrest, apoptosis	p53, p21, Bax, Fas (L), NF-*κ*B	Liver cancer	[23]
	G1 phase arrest, apoptosis	p53, p21, Bax, Fas (L)	Lung cancer	[24]
	Inhibition of invasiveness and metastasis	MMP-2, MMP-13, TIMP-2	Liver cancer	[25]
	Autophagic cell death	CAMKK, AMPK, mTORC	Apoptosis-resistant cancer	[26]
	G1 phase arrest, apoptosis	p53, p21, Bax, Bcl-2	Undifferentiated thyroid cancer	[27]
	G1 phase arrest, apoptosis	p53, p21, Bcl-2, cytochrome c	Prostate cancer	[28]
	Apoptosis	STAT3, HIF-1a, Cox-2	Liver cancer	[29]
	Apoptosis	JNK, p38, p53, cytochrome c	Melanoma	[30]
	Inhibition of invasiveness, metastasis, stemness	GSK3*β*, Wnt/*β*-catenin	Prostate cancer	[31]

**Table 2:** Therapeutic strategies for improving sensitivity to chemotherapy and radiotherapy with the use of saikosaponins in cancer therapy. Related references are listed. DOX: doxorubicin; VCR: vincristine; PAC: paclitaxel; CDDP: cisplatin; ET: etoposide; IR: ionizing radiation; GSH: glutathione; MDA: malondialdehyde.

Saikosaponins	Therapy	Mode of action	Target molecules	Cancer model	Ref.
SSa	DOX, VCR, PAC	Apoptosis	P-gp	MDR breast & liver cancer	[32]
	CDDP	Apoptosis	ROS	Cervical & ovarian & lung cancer	[35]
SSb	ET	Apoptosis, DNA damage	NF-*κ*B	Melanoma	[37]
SSd	DOX	Cytotoxicity	P-gp	MDR breast cancer	[33]
	DOX	Cytotoxicity	P-gp	MDR breast cancer	[34]
	CDDP	Apoptosis	ROS	Cervical & ovarian & lung cancer	[35]
	TNF-*α*	Apoptosis	NF-*κ*B	Cervical & liver cancer	[36]
	IR	G1 phase arrest, apoptosis	p53, Bax, Bcl-2	Liver cancer	[38]
	IR	Apoptosis	HIF-1*α*, p53, Bax, Bcl-2	Liver cancer	[39]
	IR	DNA damage	GSH, MDA	Liver cancer	[40]
